# Transitioning from crates to free farrowing: A roadmap to navigate key decisions

**DOI:** 10.3389/fvets.2022.998192

**Published:** 2022-11-14

**Authors:** Emma M. Baxter, Vivi A. Moustsen, Sébastien Goumon, Gudrun Illmann, Sandra A. Edwards

**Affiliations:** ^1^Animal Behaviour and Welfare, Animal and Veterinary Sciences Group, Scotland's Rural College, Edinburgh, United Kingdom; ^2^SEGES Innovation, Aarhus, Denmark; ^3^ETH Zurich, Animal Physiology, Institute of Agricultural Sciences, Zurich, Switzerland; ^4^Department of Ethology, Institute of Animal Science, Prague, Czechia; ^5^Faculty of Agrobiology, Food and Natural Resources, Czech University of Life Sciences Prague, Prague, Czechia; ^6^School of Natural and Environmental Sciences, Newcastle University, Newcastle upon Tyne, United Kingdom

**Keywords:** pigs, free farrowing, temporary crating, design, welfare, management, economics, environment

## Abstract

There are animal welfare concerns about the continued use of permanent crating systems for farrowing and lactating sows, which is the most prevalent maternity system in global pig production. Greater societal attention in recent years has culminated in changes (or proposed changes) to regulations as well as market-driven initiatives to move away from crated systems. Transitioning from farrowing crates to systems that allow the sow greater freedom of movement and behavioral expression requires a number of key decisions, with various trade-offs apparent when trying to balance the needs of different stakeholders. This review discusses these decisions based on common questions asked by farmers, policy makers and other stakeholders when deciding on a new system to build/approve. Based on the latest scientific evidence and practical insight, decisions such as: whether to retrofit an existing barn or build a new one, what spatial dimensions are necessary per sow place, whether to adopt free farrowing or temporary crating, how to provide substrate/enrichment and be hygienic and environmentally friendly, and how to optimize the human inputs and transition between systems are considered. The aim of this paper is to provide a roadmap for those interested in uptake of higher welfare systems and practices, as well as to highlight areas requiring further optimization and research.

## Introduction

Close confinement systems, such as farrowing crates for periparturient and lactating sows, have known animal welfare detriments and raise ethical concerns due to the physical and behavioral restrictions they involve (1). Though there have been societal concerns over their use for more than 30 years, farrowing crates are only prohibited in three countries—Sweden (since 1987), Switzerland (since 1997) and Norway (since 2000). However, legislative plans to phase out their permanent use have been enacted in Austria (by 2033) and Germany (by 2036). Though no regulations exist on farrowing crates in the rest of Europe, a recent European Citizen's Initiative (ECI) to “End the Cage Age” (2) was debated in the European Parliament in June 2021. Subsequently the European Commission stated that “by the end of 2023, a legislative proposal to phase out, and finally prohibit all cage systems would be in place,” with possible implementation as soon as 2027. They stipulated that this would follow an appropriate transition period, after a robust scientific and impact assessment [since partially completed—(3)] as part of their “fitness check” of Council Directive 98/58/EC (the current animal welfare regulations). Outside Europe, only New Zealand has committed to phasing out farrowing crates (by 2025). However, in the USA, Proposition 12 (Californian ballot approving the “Farm Animal Confinement Initiative,” 2018) highlights the trend for greater debate amongst various stakeholders about the use of confinement systems, albeit with the current focus on the use of gestation stalls. Table 1 summarizes relevant text excerpts from regulations in countries restricting farrowing crate use.

**Table 1:** Regulations for minimum farrowing and lactation standards in countries restricting farrowing crate use.

**Country**	**Date enacted**	**Minimum space requirements per sow and litter place**	**Temporary crating (TC) permitted?**	**Nest-building substrate?**
Sweden SJVFS 2010:15 (L 100)	1987	6.0 m^2^ (including a creep) with a minimum lying area for the sow of 4.0 m^2^. 75% of the lying area must be a “non-draining” floor.	TC only “for sows that are aggressive toward their piglets or show abnormal behavior that presents an obvious risk to the piglets can be confined” Length of time: Not stipulated but states “before farrowing, sows and gilts shall be able to move freely in the farrowing pen, so that they can perform nest building”	Yes: Sows must have access to “litter that enables them to perform nest-building”
Switzerland Swiss Federal Council. 2008. Animal Protection Ordinance 455.1 (Updated March, 1st 2018). (Article 50 and annex 1 - Table 3) and Ordinance on keeping of livestock and pets 455.110.1 (Updated March, 1st 2018). (Article 26).	1997 (with 10 year transition)	“Farrowing pens must be designed in such a way that sows can turn around freely” Built after 2008: 5.5 m^2^ with at least 2.25 m^2^ allocated to the sow lying area. “a contiguous lying area of at least 1.2 m^2^ with a minimum width of 65 cm and a minimum length of 125 cm must be in place in the area accessible to the sow. The minimum width of farrowing pen is 150 cm. Pens that are narrower than 170 cm must not have any installations in the rear 150 cm of the pen.” Built before 2008: 4.5 m^2^	TC in isolated cases: “During the parturition phase, sows may be restrained in isolated cases, if they are savaging the piglets or if they have limb problems” Length of time: Only “during the parturition period” which is defined as “from the beginning of the nest-building period until the end of the 3rd day following birth.”	Yes: “Sufficient long straw or other material suitable for nest building must be provided in the pen several days before farrowing and sufficient litter must be provided during the suckling period.” “Suitable” has to be something that can be carried “by the snout” not chopped straw, not sawdust but long-straw
Norway Article 11 in the regulation for conventional Norwegian pig production (Regulation for keeping of pigs, 2004)	2000	6.0 m^2^	TC only if sows are aggressive Length of time: Maximum of 7 days post-farrowing.	Yes: “pigs should have continuous access to an ample amount of materials which they can explore and be occupied. Materials like straw, hay, sawdust, peat and earth can be used”
New Zealand MPI Discussion Paper 2022/05 (Changes to the Code of Welfare for Pigs and associated regulations | NZ Government (mpi.govt.nz))	2025 (announced 2021)	6.5 m^2^ “The farrowing pen must be at least 6.5 m^2^ in total with 5.0 m^2^ for the sow.” Option A: Free Farrowing “Accommodation for farrowing and lactating sows must be of suitable design and sufficient size to allow for separate lying/nesting, dunging and feeding areas.” “Sows must be able to turn around and lie down at full length and without leg restriction.” Option B: Temporary Crating	Under Option B TC is permitted. Length of time: 72 h “If sows are to be confined in farrowing crates: i) they must only be confined after the nesting period; and ii) they must not be confined for longer than 72 h after completion of nesting behavior”	Yes: “The sow must be provided with at least 2 kg of long-stemmed straw or an equivalent volume of an alternative substrate with similar properties (manipulable, destructible, chewable) not < 48 h before expected farrowing. The flooring in the lying/nesting area must be suitable for containing the nesting material.”
		“When in a farrowing crate, the sow must be able to avoid all of the following: touching both sides of the crate simultaneously, touching the front and the back of the crate simultaneously, and touching the top of the crate when standing. When not in a farrowing crate, accommodation for farrowing and lactating sows must be of suitable design and sufficient size to allow for separate lying/nesting, dunging and feeding areas. When not in a farrowing crate, the sow must be able to turn around and lie down at full length and without leg restriction.		
Austria Tierhaltungsverordnung verlautbart (ThVO), Federal Law Gazette II No. 485/2004; amended by Federal Law Gazette II No. 61/2012	2033 (announced 2010)	≥5.5 m^2^ 50% lying area with 1/3 solid floor (max. 5% openings) “Room for free movement for sow”	Yes: “Crating only in critical period of piglet's life”	Nothing above EU regulations
Germany Tierschutz-Nutztierhaltungsverordnung in der Fassung der Bekanntmachung vom 22. August 2006 (BGBI I.S. 2043) die zuletzt durch Artikel 1a der Verordnung vom 29. Januar 2021 (BGBI, I.S. 146) geāndert worden ist https://www.gesetze-im-internet.de/tierschnutztv/BJNR275800001.html *Unfallverhütungsvorschrift Tierhaltung https://www.agrarheute.com/media/2021-03/unfallverhuetungsvorschrift-tierhaltung-svlfg.pdf	2036 (published regulations in 2021)	6.5 m^2^ “the floor area must allow the gilt or sow to turn around freely” “…the lying area for gilts and sows must be designed in such a way that that the degree of perforation does not exceed 7%.”	Yes: Length of time: Maximum of 5 days “Gilts and sows may only be kept in the crate for a maximum period of 5 days, which includes the time in which the gilt or sow farrows” There is also a statutory requirement within additional regulations for keeping pigs (*Unfallverhütungsvorschrift Tierhaltung—Accident prevention regulation for animal husbandry) that states: “The operator must ensure that [...] farrowing pens are designed in such a way that no hazards can arise from the mother sow when catching or treating the piglets”	Yes: “…every pig has access at all times to organic and fiber-rich employment material which is safe for health and is available in sufficient quantities, which: a) can examine and move the pig and b) can be changed by the pig and thus serves the exploration behavior”
EU Council Directive 2008/120/EC	1997	No restrictions on farrowing crate use. “An unobstructed area behind the sow or gilt must be available for the ease of natural or assisted farrowing” “Farrowing pens where sows are kept loose must have some means of protecting the piglets, such as farrowing rails”	Farrowing crates permitted	“In the week before the expected farrowing time sows and gilts must be given suitable nesting material in sufficient quantity unless it is not technically feasible for the slurry system used in the establishment”

The EU regulations are presented for reference (shaded final row). The table has been produced based on the authors' knowledge of existing regulations at the time of publication.

Research into the welfare detriments associated with farrowing crates, biological needs of sows and piglets, and designs of alternatives has been extensively reviewed (4–8). Some publications have highlighted benefits of using higher welfare farrowing and lactation systems and practices (hereafter referred to as alternatives), including benefits for piglet growth and cognitive development (9–11), and for sow hormonal status, ease of farrowing [for review—(12)] and colostrum quality (13). As well as scientific evidence, there is a growing population of farmers that report positives when transitioning from conventional crates to one of the many alternatives available. These include higher weaning weights and calmer sows, including upon remixing post-weaning (14). Although some articles have reported higher piglet mortality in loose systems [e.g., see review (6)], the concern about substantial increases in mortality of piglets when the sow is not permanently crated has not always been realized by those who have made the transition (14, 15). Although there are still challenges, both practically and politically, hearing that the critical element of piglet mortality is not insurmountable gives greater confidence to other farmers considering changing to higher welfare alternatives. It is well-established that peers serve as important sources of information and can affect adoption of new technologies (16). Those pig farmers who have already adopted new systems are an invaluable source of information for others considering change (17), and their practical insights will contribute to this review. Understanding more about general motivation for change could also help with transitioning to new systems or practices. Anneberg and Sørensen (18) highlight three aspects that are important for farmers as motivation for change. One is structural change, including legislation, market-driven initiatives, audits and supplementary payments. A second includes personal competences and values, either for the farmer or amongst stockpeople. This includes the freedom to make decisions and attitudes to animal welfare. The third is the social life at and around the farm, including professional networks with colleagues, advisors and veterinarians, and involves benchmarking as motivation, as well as the collaboration between the farmer and stockpeople (18).

**Figure 1:**
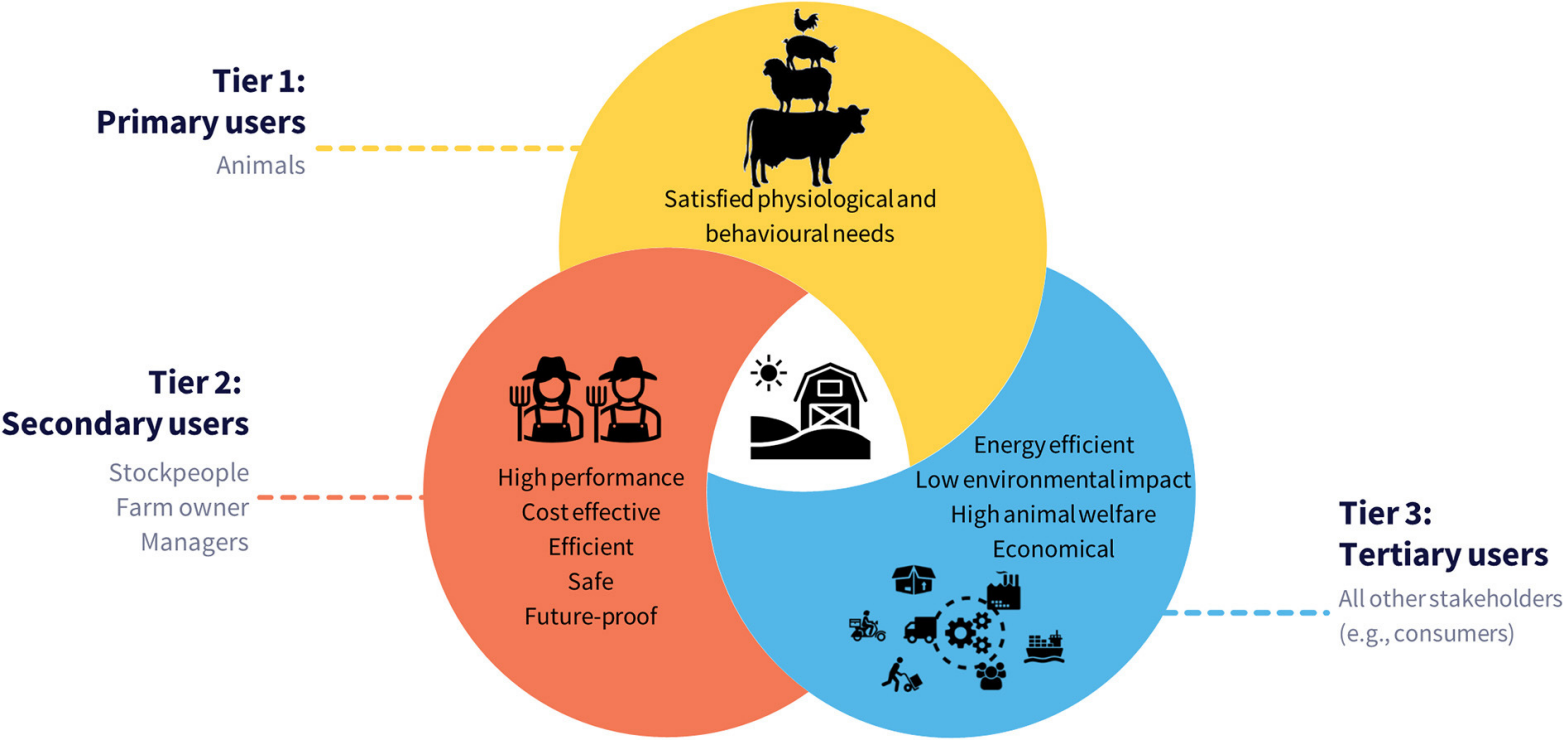
Diagram representing the “triangle of needs” between all users of a farming system. Tier 1 involves the animals who are the primary users occupying the system 100% of the time. Tier 2 are secondary users who are on the farm daily (e.g., stockpeople). Tier 3 are tertiary users and includes all other stakeholders who have an opinion about the system (e.g., consumers, retailers, and civil society) but may never interact directly with the system.

Whilst research has extensively studied certain aspects of alternative systems/practices, knowledge gaps remain, particularly on very specific design details, such as space needed for sows to turn around, and on potential long-term benefits for sows and piglets from high-welfare alternatives. Despite these gaps, there is enough information to allow translation of the evidence-base into design components of suitable pens that meet the biological needs of the animals. However, there are competing needs (Figure 1) from other users (owners and stockpeople) and influencers (supply chain, wider society) of the system. Beyond welfare goals, a system must be manageable, economically viable, sustainable and socially acceptable. Achieving all of these elements is challenging and trade-offs are inevitable. However, acknowledging these competing needs is important and will help avoid costly mistakes akin to those experienced by the poultry industry in the transition away from battery cages (19).

Figure 1 presents the “big picture” when establishing any new farming system; it must meet the “triangle of needs” for each user of that system (4), with the primary users (Tier 1—Figure 1) being the animals occupying the system. Secondary users (Tier 2—Figure 1) are the next heaviest users of the system, particularly the stockpeople. Tertiary users (Tier 3—Figure 1) represent all other external stakeholders. Though they may never interact with the system directly, they can be highly influential in its design and uptake.

Regarding “Tier 1,” the importance of meeting animal needs to maximize biological fitness, and the behavioral and physiological needs of pigs around the time of farrowing and lactation, have been discussed elsewhere (1, 4, 20). Tiers 2 and 3 have received less attention in scientific studies, yet they are key to decision making. Here we attempt to synthesize the complexities within and between each tier and provide a roadmap for transitioning from confinement in a crate throughout farrowing and lactation to greater freedom for the sows.

## Free farrowing or temporary crating?

Using appropriate terminology is important to distinguish different designs and management practices, as well as to ensure transparency and encourage consumer trust (21). The term “free farrowing” (FF), which should indicate no use of a crate to confine the sow, is often used as a catch-all term describing any alternative to the farrowing crate. However, in many alternatives, sows are crated and not able to freely turn around during parturition. The most common alternative available commercially, and in operation in countries without regulations prohibiting farrowing crates, is the temporary crate [TC; (7)]. The majority of TCs involve the use of a modified farrowing crate intended to allow the sow to be loose pre-farrowing, before restraining her during farrowing, then opening the crate (typically at 3–7 days post-farrowing) to allow the sow greater movement possibilities for the rest of lactation. These TC systems range in spatial footprint from the same size as pens with conventional farrowing crates (3.6–4.6 m^2^) to larger systems (5.5–7.4 m^2^). Appropriate synonyms for these TC systems are *loose lactation, free lactation* or *temporary confinement*. Even in countries prohibiting crating there are caveats within the regulations, permitting short periods of confinement for some situations (e.g., “aggressive sows”—Table 1). However, these countries have adapted over decades to sows being loose in these systems and the majority operate zero-confinement/crating (i.e., true FF). There are a variety of FF pens ranging in size and complexity, but the main common feature is the absence of a crate to confine the farrowing sow (although some systems may incorporate a feeding stall to aid management).

Other alternative systems include group or multi-suckling systems (22). These refer to a practice where indoor sows are either (i) grouped together before farrowing and have individual, voluntary access areas in which to farrow, or in which they may be confined for a short period around farrowing, or (ii) farrow individually in either crates or pens and are then relocated and mixed into a group with other sows and litters, typically after 10–14 days (i.e., multi-suckling). Finally, there are outdoor systems that operate as FF in huts within individual or group paddocks (1).

Each system then has extra levels of design details. The design features and inputs determine how effective a system is in meeting the animals' needs (e.g., space for turning, flooring for substrate provision, separate microclimate for piglets), stockperson's needs (e.g., protection from sows during husbandry procedures) and in addressing other externalities (e.g., environment—suitable flooring for minimizing emissions). These details can have a large influence on system performance as well as cost.

## What are the key decisions when designing a new indoor farrowing system?

**Figure 2:**
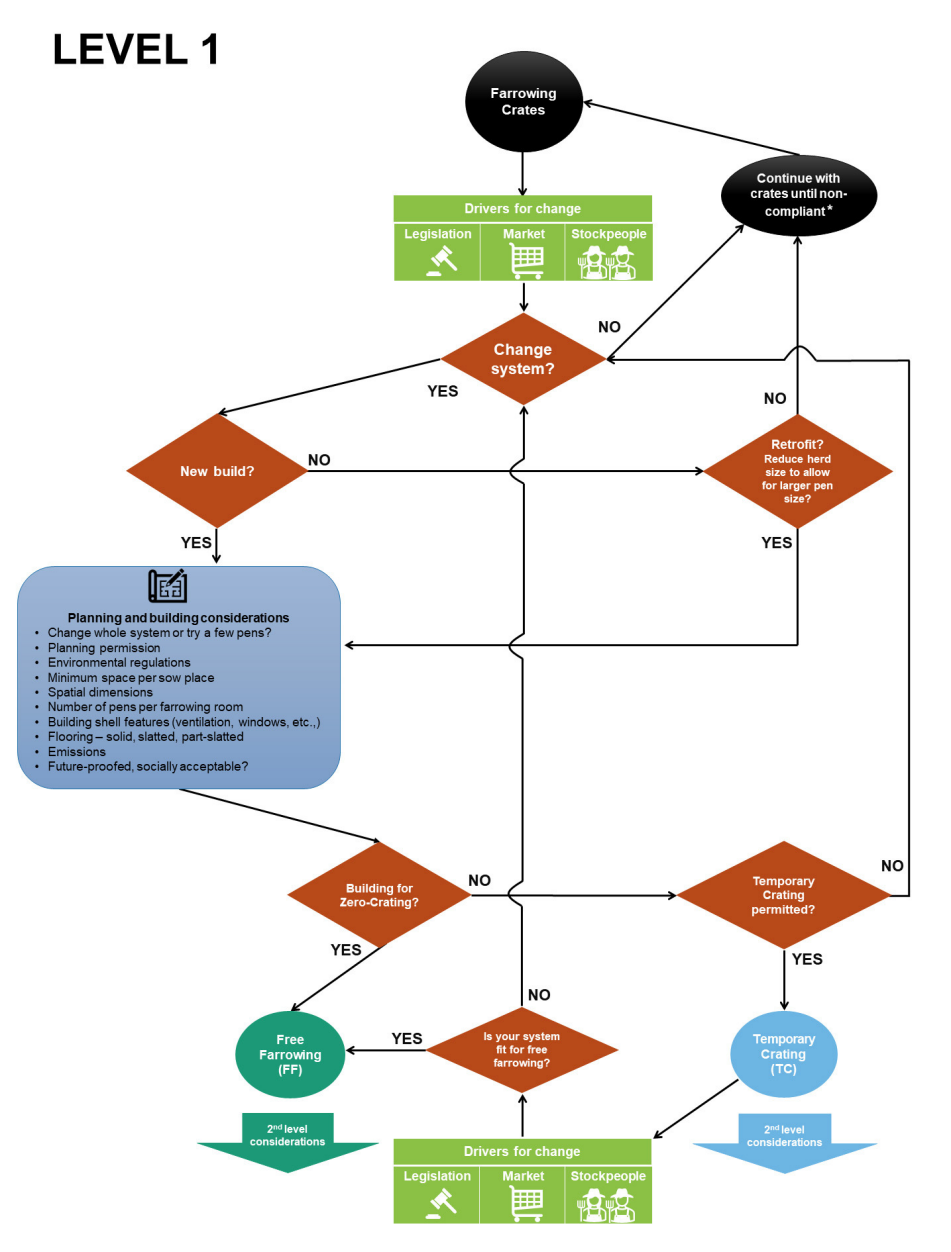
First level decision tree for transition from farrowing crates to alternative accommodation. *Assumes a transition period will be in place before farrowing crates are no longer permitted. Note that it is possible current systems may become non-compliant based on current genotypes not just permanent confinement. This is because hyperprolific sows and their litters may not be able to be accommodated in standard farrowing crate and pen sizes.

**Figure 3:**
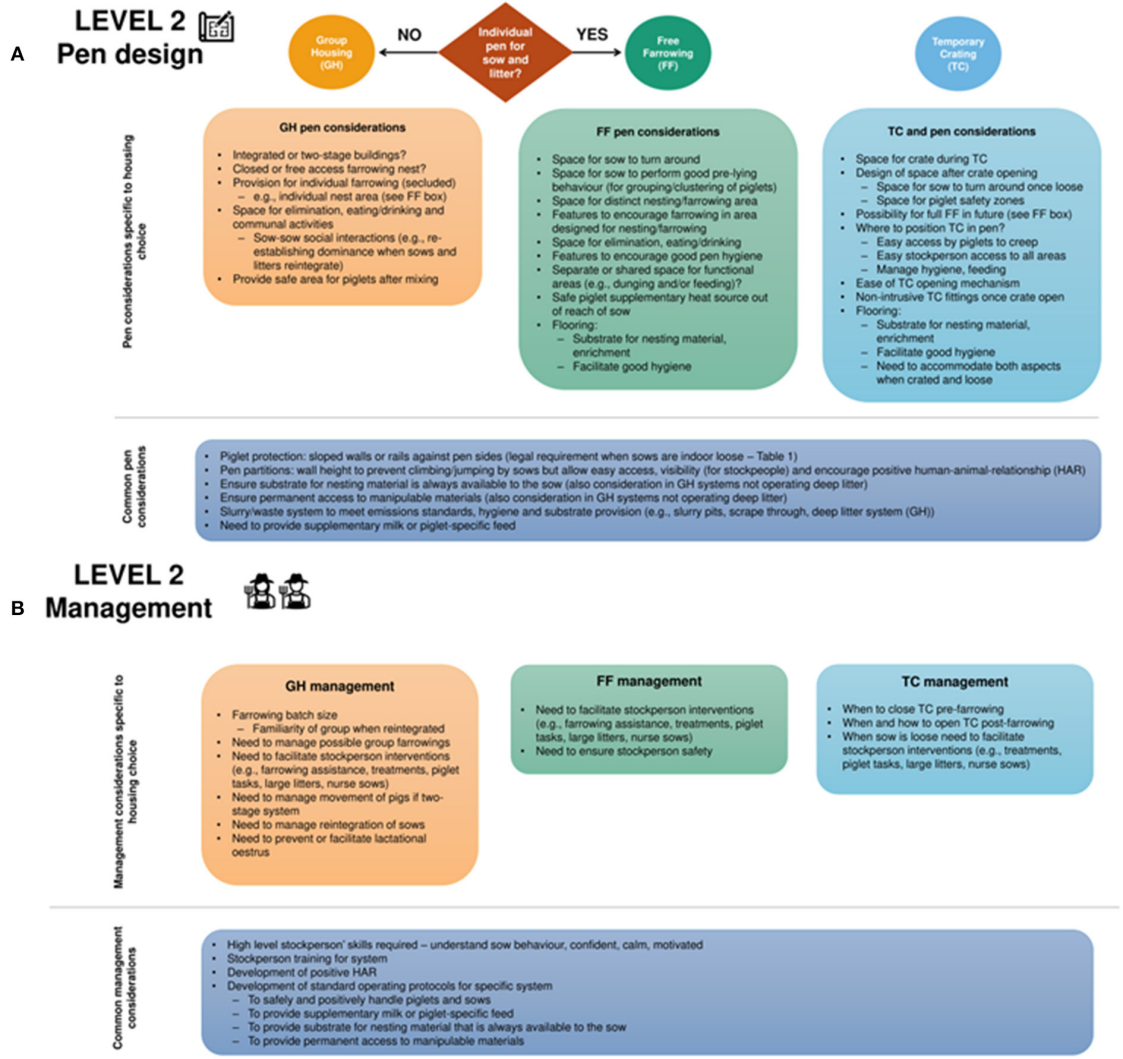
Level 2 pen **(A)** and management **(B)** considerations once a housing system has been chosen. Specific considerations for FF (free farrowing), TC (temporary crating), and GH (group housing) are given in individual boxes. Cross-referral between boxes is indicated in the text. Some considerations are common to all systems.

**Figure 4:**
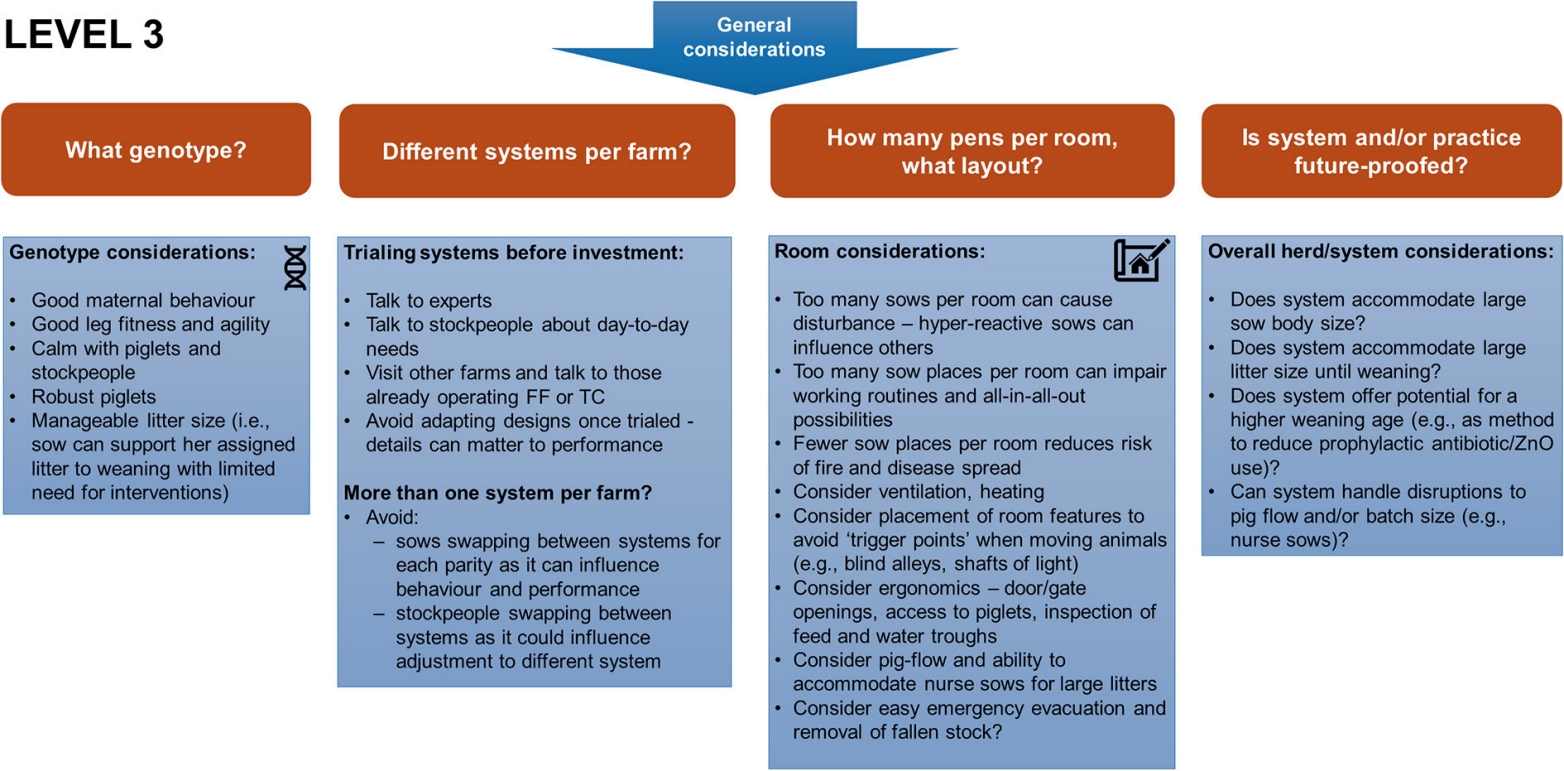
Level 3 shows the general considerations when deciding on a new system and implications of choices. Questions should be asked about suitable genotype, whether more than one housing system will be on farm, the overall pen and room layout and whether the system and/or practice is fit for now or the future.

Multiple decisions are required when changing to, or establishing, a new system. Figures 2–**4** present a series of flow diagrams of the key considerations in this decision-making process. They depict the process as multi-layered, with the first layer involving top-level and often irreversible decisions that are likely to be influenced by externalities such as regulations on animal welfare, environment and planning (local, national and international) and/or market-drivers, as well as finances (Figure 2). If there are impending legislative and/or market-drivers to abolish farrowing crates, farmers can either operate crates until they are no longer compliant or choose to change system. Changing system will involve retrofitting an existing barn or building a new one. Any new or retrofitted system will require more space per sow to allow for greater movement. The next major question is whether any new system will retain the ability to crate the sow, even temporarily. These factors influence planning and building considerations.

The second layer considers the details that are required when choosing a pen design within each category of system (Figure 3A) and includes important considerations for management (Figure 3B).

The third layer summarizes general considerations for other inputs, such as sow genotype (Figure 4). These figures demonstrate the complexities involved in changing systems, with much discussion and detail required when considering the various trade-offs that inevitably occur when trying to satisfy multiple stakeholders and future-proof for potential externalities (e.g., further changes to legislation).

### Building for now or the future? Retrofit or new build?

Retrofitting barns may seem cost-effective and sometimes might be the only option as a result of finances and/or restricted building opportunities [e.g., see German regulations; (23)]. However, it could be a false economy if the space is sub-optimal for the choice of pen design, genotype and for establishing efficient day-to-day working routines (Section Human-animal relationship). A typical farrowing crate occupies 1.2–1.4 m^2^ within an overall pen spatial footprint of 3.6–4.6 m^2^ (8, 24–26). Simply removing the crate and using this space for FF or operating it as a swing-side TC option, generally results in higher piglet losses (3, 7, 27, 28) because the sow cannot easily exhibit piglet avoidance behaviors when changing posture. Sows and litters are now bigger than when many current crate systems were built (29–31). Meyer (30) concluded that the German legal spatial requirements for farrowing crates of 2.0 × 0.70 m no longer met the sow's space requirements due to her increased size. Furthermore, in loose systems, more space is required to perform different functional behaviors, such as elimination, whilst maintaining hygiene.

Most farrowing buildings were designed for a lifespan of ~20 years [based on a typical depreciation period, (32)]. However, many have existed beyond this period (33) with subsequent outlay covering running repairs but no major investment. Given the significant investment required to transition to a new system, and the tendency for systems to remain unchanged once built, it is sensible to establish a system that is fit for the long-term future, not just for an interim period. Regulations could change and so could consumer demands; in particular, TC may be acceptable in the interim but may not be future-proofed (Section Societal acceptability). What we do know now is that space per sow place must increase and therefore any retrofit necessitates a reduction in overall herd size, which (all other things being equal) impacts farm profitability. A new-build scenario, which might include rebuilding an old barn or adding extra sections, is the most future-proof option, and with that scenario comes a list of planning and building considerations (Figures 2, 3), many of which start with questions about space requirements. If farmers choose a hybrid option of both rebuilding/retrofitting an old barn and also building new there is a risk of ending up with two different designs as pens in the retrofitted barn maybe subject to constraints of that older barn. Having two different designs could influence optimization of management as stockpeople and sows experience different systems (see Section The learning curve associated with a new system).

### Single or group housing?

Group housing (GH) during lactation can seem like an attractive alternative, being cheaper in investment than indoor single housing (34). Indoor group housing obviates the need to consider land choice (i.e., soil type) and outdoor climatic conditions, which are both major factors determining the feasibility and success of outdoor systems. However, group systems present greater management challenges (Figure 3B), including potential for farrowing disturbance by other sows, desertion of litters, cross-suckling by piglets, difficulties in feed control and occurrence of uncontrolled lactational oestrus which disrupts batch management [see reviews of (8, 22)]. Some GH systems/practices have recently been investigated with the aim to reduce weaning stress by employing protocols to encourage controlled lactational oestrus, thus prolonging suckling, however such systems require very careful management (35). The case study of Li et al. (36) illustrated the generally higher and more variable performance in group systems and their dependence on correct management. As a result of these considerations, group systems are relatively uncommon in commercial practice.

### How much space?

Space is often the starting point for new building decisions; it dictates finances, planning permission, herd size and it impacts on animal welfare, labor demands and emissions potential (involving concerns of all three stakeholder' tiers; Figure 1).

Animal welfare regulations often specify minimum spatial requirements, in part because such metrics allow relatively easy inspection of compliance. Thus, in countries with regulations prohibiting farrowing crates now or in the future, stated minimum farrowing pen sizes range from 5.5 m^2^ in Switzerland to 6.5 m^2^ in Germany (Table 1). However, these minimum footprints are likely to require TC for effective operation (7). If the intention is to operate true FF, 7.0 m^2^ is considered the likely minimum space for successful operation. This value was suggested as early as 1992, to be the minimum area for a loose farrowing pen that allows “*sows and piglets to behave appropriately to their species*” (37). This is further elaborated in a review of performance of loose systems, which suggests that important piglet gathering and grouping behaviors by sows influence the minimum space needed (38). In their recent opinion on pig welfare, EFSA have stated that 7.8 m^2^ is the recommended minimum pen size for FF with 6.6 m^2^ available to the sow (3). This is to achieve the same piglet mortality in a FF system as a permanent crate system, but they also state a 90% certainty range from 5.7 to 11.0 m^2^ because of the important interacting effects of design detail and quality of management. When interviewing/surveying farmers from countries with FF as standard, they regularly say that their minimum space regulations are not enough and typical pen sizes in operation are larger [e.g., 7.0 to 8.3 m^2^ in Switzerland and Norway (39, 40). Andersen and Ocepek (40) comment that the larger pen space in Norway is, in part, due to the future trend of building “from-farrowing-to 30 kg pens” where the piglets can remain and stay with their litter mates after weaning and the sow is moved. This reduces aspects of weaning stress and could improve the resilience of piglets without the use of post-weaning additives (e.g., Zinc Oxide) and antibiotics, whose prophylactic use was common in the past but is now prohibited in the EU (41).

## Tier 1 stakeholder needs and considerations

The minimum spatial dimensions (i.e., width and length) necessary for a system to work successfully are dictated by the needs of the Tier 1 users–the animals.

### How should minimum space needs of animals be determined?

In their review, Goumon et al. (7) point out that space is not only a question of area, but also dimensions to accommodate the length, width, height, and locomotion of sows and piglets (Section Pen shape and layout). Therefore, minimum pen spatial dimensions should be determined by the number of occupants, their body dimensions and how they move dynamically in order to carry out functional (particularly highly motivated) behaviors. The body dimensions of modern crossbred sows were measured on Danish farms by Moustsen et al. (29), who suggested a significant increase in sow size over the preceding years, and since repeated by Nielsen et al. (31) to determine if there were any further increases. Though there were seven years between the two studies, no increase in sow dimensions were observed, neither for younger nor for full grown sows, suggesting that sow size has stabilized. Using these data Baxter et al. [(4), updated in 2018 (1)] summarized space requirements for various activities the pigs perform during farrowing and lactation.

To accommodate these requirements, the spatial dimensions need to be large enough to allow full zonation for functional behaviors (e.g., dunging separate to feeding, separate to nesting/resting) and a separate piglet creep area. A compromise might be to share space for some functions, as sows would not be eating and resting simultaneously for example, but the design detail of that space dictates how effective this compromise would be. In Goumon et al.'s review of TC (7), pen design is discussed at length and minimum space depends on whether or not TC is permitted during the neonatal period.

Covering each requirement/need is a detailed and complex process explored elsewhere [e.g., (1, 4)]. Here we will give examples of some fundamental aspects that dominate space conversations, such as accommodating sow turning behavior.

In loose systems (including TCs when operated open), turning is necessary to enable effective mother-offspring interactions. Schmid (42) observed that the sow behavior of grouping piglets before lying down becomes less successful with decreasing farrowing pen space [from 7.5 (2.5 × 3.0 m) to 4.1–5.0 m^2^; pens with swing-side TCs ranging from 1.8 × 2.3 m to 2.0 × 2.5m)—dimensions specified in (37)]. They noted that successful grouping was specifically promoted by the sow turning around repeatedly. Cronin et al. (43) found that the dimensions of the farrowing nest affected sow and piglet behavior, with smaller-sized farrowing nests [3.4 m^2^ (2.0 m depth × 1.7 m width)] compared to larger 4.3 m^2^ (2.4 m depth × 1.8 m width) resulting in reduced piglet survival. Baxter et al. (44) found that larger pens with larger nests [9.7 m^2^ in total; nest-site = 4.0 m^2^ (1.3 × 2.8 m) vs. 7.9 m^2^ in total; nest-site = 3.3 m^2^ (0.9 × 2.4 m)] resulted in higher piglet losses. Even though the nest-site had protective features against crushing, these authors suggested rolling behavior and the potential for piglets becoming chilled when born further from the heated creep as reasons for losses. These various studies, with somewhat contradictory results, highlight that important knowledge gaps remain, particularly about space for sows to safely gather piglets and hence minimize crushing, and its interaction with other nest features. The absence of a farrowing crate, or of impeding features in a small pen, will promote more successful suckling interaction, since this facilitates piglet access to the udder which can translate into increased weaning weights for piglets raised in alternatives (9, 45–47). In addition, restriction in the farrowing crate influences sow endocrine status, notably reducing oxytocin levels, that can negatively impact on piglet suckling success (13, 48).

The spatial dimensions also impact on hygiene. Bøe et al. (49) looked at pen cleanliness in several herds operating alternatives. Whilst there was much variation in pen cleanliness between herds, there was a tendency for cleanliness to improve with increasing pen size. Size was not the only factor, however; the presence of slatted flooring in the dunging area, its depth and provision of substrate also influenced pen hygiene (Section Flooring and substrate).

### Quality of space—The importance of pen features

#### Pen shape and layout

**Figure 5:**
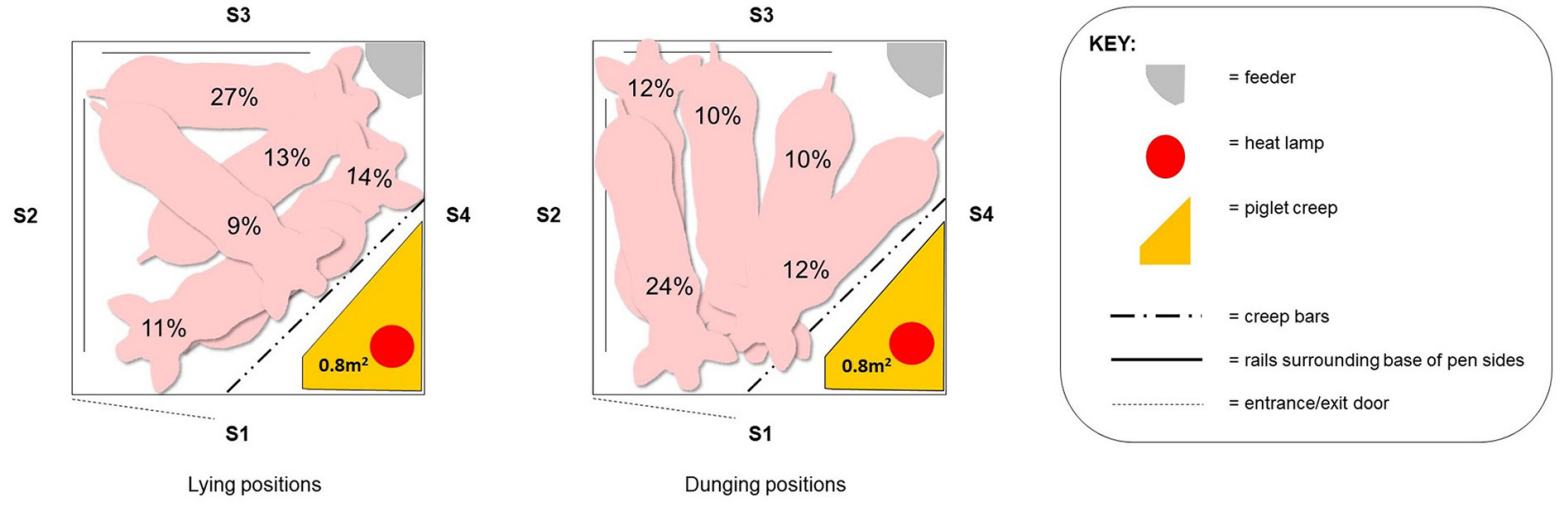
The five preferred lying positions (left) and dunging positions (right) in a square pen (2.4 × 2.4 m) when the sow was loose [adapted from Moustsen et al. (51)]. S1: Pen side at inspection aisle; S2: Pen side opposite piglet creep; S3: Pen side opposite inspection aisle; S4: Pen side/furniture in front of piglet creep.

It is not just the quantity of space that determines the success of a system. The quality of that space (e.g., components, design features, how the space is arranged into an optimum pen) is critical to satisfy all needs. When sows have the option, they will divide the pen into different functional areas, including separate areas for resting, eating and dunging (4, 5, 50). Pen width and length determine the possibilities for sows to orient when performing maternal, feeding and elimination behaviors. Pens of rectangular shape appear to offer better possibilities to maintain separate functional areas than square (equal-sided) pens; the dunging area in rectangular pens will be alongside one of the short pen sides, which increases the distance between resting and dunging areas (50). The recommendation for the dunging area is to be at least a sow-length in width and 1.0 m in depth (5). The problems with a square pen are illustrated in Figure 5 [adapted from Moustsen et al. (51)]. They observed where sows chose to lie and dung when loose in square TC-pens (2.4 × 2.4 m). Dunging and resting areas overlap as the sows have little possibility to separate these functions within pens of these dimensions. Poor pen hygiene impacts on animal health, but also stockperson time management if the flooring and dung removal system does not facilitate clean pens (52). The impact of pen layout on hygiene therefore also has implications for flooring choice (Section Flooring and substrate).

To encourage farrowing in the optimum location for piglet survival (i.e., close to a supplementary heat source, easy access for stockpeople if intervening), the pen needs features that encourage the appropriate sow choice. These include a degree of enclosure and presence of nesting substrate (4, 20). As discussed above (Section How should minimum space needs of animals be determined?) a pen may have a generous amount of space but that does not always translate into good piglet survival. Having too much space without features to promote piglet survival (Section Piglet protection features), can be detrimental. If the pen is too large for newborns to locate a supplementary heat source, it could increase the risk of hypothermia (44), depending on thermal properties of the flooring and substrate provision. Physical protection for piglets to reduce crushing risk is a legal requirement for loose systems (Table 1) but thermal protection requires careful thought about creep placement and accessibility in relation to farrowing location, the ability to supply supplementary heat sources safe from damage by the sow and the ability to provide thermally protective substrate. Having thermal differentials within a farrowing pen is considered an important part of directing both sow and piglets to perform functional behaviors within areas designated for those functions (e.g., farrowing/resting in a lying area, dunging in a slatted area) (4). Achieving such temperature differentials can be challenging in hotter climates. Ideally the environment would provide a thermoneutral zone for the piglets in the creep and be thermoneutral for the sow in the pen (i.e., cooler outside of the creep). If it is warm in all areas of the pen, neonatal piglets are more likely to lie outside of the creep, taking up more space if lying laterally to facilitate heat dissipation (53). They are then at greater risk of being overlain by the sow, particularly if she is loose-housed. Climate-controlled buildings would be recommended to facilitate temperature differentials in farrowing pens.

#### Flooring and substrate

Whilst it seems obvious that flooring should provide a non-injurious, non-slippery surface to facilitate safe movement and posture changes, there has been very little research on the effectiveness of different flooring materials in FF or TC pens (54, 55). The large difference in claw size and thermal demands between the sow and her piglets makes floor choice challenging when both must share the same area (56). The type of material used needs to support the sow's weight, being mindful of greater movement in all sections of the pen when she is loose, as well as be comfortable for both sows and piglets when resting. Good drainage is important to maintain hygiene, particularly when separate functional areas cannot be established, and for this reason fully-slatted pens are common in small TC designs (7). However, absence of solid flooring generally precludes the possibility for the sow to construct a nest, a behavior that she is highly motivated to perform, because nesting substrates, such as straw, are rarely offered if they pass through a slatted floor and impair manure management. The compromise is to design a part-slatted pen, with sufficient solid area to permit nesting and a slatted dunging area to give continuous removal of excreta. This also reduces the surface area of the slurry pits and so benefits air quality, including lower ammonia emissions (Section Environmental impacts). The problem of limited substrate provision in practice might be mitigated by adopting a building with a manure handling system, such as under-slat scrapers (54), rather than a vacuum-operated-system, but this obviously has wider whole-farm implications. At present, it is usually mitigated by providing non-particulate nesting substrate, such as ropes or jute/hessian sacks (57, 58) although the adequacy of these requires further research and technical innovation in flooring to manage multiple requirements is also needed.

#### Piglet protection features

European legislation states that there should be farrowing rails or similar to protect piglets in pens for loose sows (Table 1). Sows lying down unsupported is a risk factor for piglets being crushed and sows show a preference for sloped or vertical walls rather than rails when supporting themselves lying down (59). When placing sloped walls or farrowing rails, it is important that the dimensions and distances ensure sufficient space for piglets to escape safely behind the barriers. They must therefore provide at least the shoulder width of a 3-to-4-week old piglet. If a piglet is meant to easily go underneath a feature, the distance from floor to rail/wall should be at least the height of a newborn-to-7 day old piglet, ideally accommodating a weaner (60), but less than the body/shoulder width of a young sow to avoid them getting “stuck” between floor and rail/wall [200 mm is recommended based on available data—(31, 60)]. Similarly, creep entrances should be wide enough for piglets to access until weaning, but not so wide that sows might be able to get their heads in and/or get stuck. In 2004, Moustsen and Poulsen published dimensions of 109 Danish crossbred piglets (60), and in 2017, Moustsen and Nielsen repeated the study (61). Dimensions had changed very little over that period, with 4-week-old piglets having shoulder widths of 80–130 mm. Similar dimensions (138–143 mm) have been reported by authors in the USA measuring 150 crossbred weaners (62) and by Smith and Ramirez (63) who estimated the width of 5 kg piglets to be 110–111 mm and 10 kg piglets to be 151–153 mm. Based on these dimensions and those known about the sow, a width of 180 mm is considered optimum (60) and somewhat future-proofed for larger piglets.

For TC systems, placement of the crate in relation to structures such as sloped walls is important to ensure these do not impede piglet teat seeking and suckling. Additionally, when a TC is used, it is important to consider the space that the crate occupies when open. If it does not fully open flush with pen walls or fold up, it limits the spatial dimensions subsequently available for the sow. It is also important to ensure that the fixtures holding the crate in place when closed do not become a risk factor for injury after it is opened, especially if these protrude into the pen (64).

#### Crate opening and closing procedure

Temporary crating requires extra protocols to decide on the best operational approach to managing the crate. The choice of timing for enclosure of sows pre-partum and release post-partum has been discussed extensively elsewhere (7). The recommendation is that sows should be loose until as late as possible before farrowing and crates should be opened up on an individual litter basis (65) at 3–5 days after farrowing (7), although EFSA's recent opinion (3) suggests that a minimum of 7 days is required for a TC with an average space for the sow of 4.3–6.3 m^2^ to achieve the same piglet survival as a permanent crating system. However, this estimate had a 90% certainty range of 3.4–16 days, reflecting the variation associated with detail of system design and management. For certain assurance schemes earlier opening is required if TCs are permitted at all (Section The market for meat).

Allowing sows to be loose before farrowing is important to permit the different aspects of nest-building behavior, including the increased ambulation sows undertake as part of nest-site seeking which occurs as early as 3 days pre-partum (66). Given the absence of piglets at this stage, there is no reason to enclose the sows, and allowing the sow to nest-build benefits both her and her piglets post-partum *via* improved maternal behavior, oxytocin and colostrum quality (67). Correct timing and procedure to close the crates is important to reduce the risk of disturbance and stress around farrowing. Interrupting nest-building to crate before farrowing may be undesirable, but interrupting once farrowing has commenced can have detrimental consequences, including increasing farrowing duration (48, 68). This is discussed at length elsewhere (3, 7).

#### Pen partitions

The design of pen partitions impacts on maternal behavior and how sows react to stockpeople. If pen partitions are too low, sows can climb/jump and the feeling of enclosure required to promote correct farrowing location in the nest is reduced (4). However, if they are too high and it is difficult for stockpeople to inspect pigs and feed troughs from outside of the pen, or exit easily when inside the pen, it can impact on time management (52) and safety (Section Tier 2 stakeholder needs and considerations), as well as effectiveness of tasks such as farrowing supervision. High walls can also increase fearfulness in piglets (69) and therefore potentially the maternal defensiveness of sows. Gortz et al. (70), after trialing five different FF and TC designs with wall heights of 0.9 and 1.0 m, recommended a minimum height of 1.0 m for walls that the sow has access to; below this height, there were issues with sows climbing. However, Hales et al. (25, 71) used wall heights of 0.9 m and no incidents of sows climbing were reported. This would likely be the minimum height based on the physical dimensions of modern sows (29, 31). Introducing additional partitions within the pen can help delineate the lying and dunging areas (72). It is also important to recognize that whilst solid partitions are important to create a sense of enclosure and improve pen biosecurity, they can alter air-flow and could negatively affect thermal comfort and pen hygiene (73).

### Room design and pen number per room

Much attention has been given to the specific features of pen design required to optimize performance and meet biological needs (4). In contrast, little attention has been given to the room design as a whole regarding ease of management (Section Tier 2 stakeholder needs and considerations) and impacts on animal welfare and health. Pen positioning within the room, correct matching with the ventilation system, access around the pens and number of pens in a room can have a considerable impact on success of systems. Too many pens per room (Section Work conditions and room layout) can be disruptive, especially if there are sows that may be hyper-responsive to their environment. This relates to the social contagion effect, whereby pigs will react to each other's positive and negative experiences (74). There may be other “trigger points” in a building; entrance and exit ways into rooms/pens may have hazards impairing ease of movement, risking both animal and stockperson welfare. For example, if entry to pens involves approaching a blind alley a sow will be hesitant. Pigs have wide monocular vision but are not good at short-range vision, so sudden changes in lighting that show up on the floor could cause a sow to freeze; pigs may perceive shadows and shards of light as changes in floor surface (75). So, whilst natural lighting in buildings is considered beneficial for stockpeople and animals and is looked upon favorably by consumers, placement of windows and the direction of sunlight may have unintended impacts on moving animals through buildings and should be considered when deciding on overall building design and placement of features in the building shell.

### Sows and systems

In the discussion to date the sow has been considered as a generic entity, however sows show individual differences in behavior and this needs to be accommodated in all systems. Behavioral traits which lead to successful offspring survival in FF systems, and have been shown to have a genetic component, include lack of aggression toward piglets, calmness or lack of fearfulness, carefulness around piglets, responsiveness to piglet distress and good nursing behavior [for review (76)]. Another desirable trait in FF sows is lack of aggression toward people, since stockperson safety is an important consideration (Section Stockperson safety and time management). Recent work (77, 78) developed tests characterizing the behavior of lactating sows toward humans in alternative systems. Behavioral traits derived from these tests could be used as new phenotypes for the genetic selection of gentle and easy-to-handle sows. Whilst selective breeding for traits which fit FF systems may help at the population level, this process is still at a very early stage of implementation and most sows from nucleus herds still farrow in crated systems. Genotype-by-environment interactions are likely to influence many important traits and there will still be individual differences, exacerbated by the previous experience of the sow (79). These can be mitigated by handling protocols promoting a positive human animal relationship (HAR; Section Human-animal relationship). Adopting a positive HAR should facilitate the large number of interventions required to promote piglet survival, particularly with large litters (80). However, to mitigate the need for such interventions, which are more challenging to perform in alternative systems, selection programmes should consider more manageable litter size, more robust piglets and an emphasis on sows rearing their own litters.

## Tier 2 stakeholder needs and considerations

Tier 2 stakeholders include the stockpeople who work in the system on a daily basis, and those responsible for the investment and management choices which are made. The consistent production of large litters requires significant interventions by stockpeople to promote piglet survival and this is perhaps one of the major barriers to adoption of a truly FF system (along with concerns about costs, not only of installation but for long-term production). The crate has advantages in controlling sow movement (to reduce crushing), allowing localized heating at the birth site, and facilitating safe targeted interventions to promote piglet survival such as assisted suckling, split suckling and cross fostering (Section Work conditions and room layout). These interventions are more commonplace when working with highly prolific genotypes and pose challenges which, although present in all systems, may be particularly important in FF systems. Sows are most protective of their piglets in the first few days post farrowing (81), the period when the majority of piglet handling by stockpeople for routine husbandry tasks and/or interventions to promote piglet survival takes place, constituting one reason why systems that permit use of a TC are more popular. This is recognized in Austria and Germany where “crating during the critical period for piglet survival” is permitted within their legislation (Table 1) and in Denmark [renowned for large litters—average born alive 17.7—(82)] where loose lactation is emphasized (83). It is therefore not surprising that the majority of more recent research has had a focus on TC (7).

Management is just as critical as pen design, if not more so, in making alternative systems successful. That was the message at a workshop on Freedom in Farrowing and Lactation (14) where the theme was “Overcoming barriers, facilitating change.” Throughout the workshop, discussion returned to aspects of management, including feeding, daily routines, health, timing of measures such as confinement and handling of sows. Routines in farrowing crates are well-established and fairly generic, but the variety of alternative systems means that a given system and accompanying husbandry protocol may work well on some farms but not others. Farrowing systems that give the sow greater freedom of movement constitute a big change for stockpeople experienced with established protocols for crates. Some procedures must be managed differently and will require greater attention to animal behavior (including developing a positive HAR) in order to carry out routines safely and effectively. Time management will be dependent on the specific system and design features that aid working routines.

### Stockperson safety and time management

Stockperson safety is a high priority in any workplace. There are valid concerns when sows and stockpeople share the same space and, in some countries, there are additional regulations regarding stockperson safety specifically around farrowing sows (e.g., Sweden, Germany—Table 1). There are specific features of a system that will impact on stockperson safety and time management. Some have been formally researched, others are reported from the gray literature including farmer testimonials at workshops, case studies and in interviews.

#### Animal handling

To reduce the requirements for entering the pen, which can be both a safety and a biosecurity risk, design features facilitating management of routine tasks from outside of the pen are recommended. The most common reasons stockpeople need to enter the pen are for cleaning (Section Multisystem comparison studies) or to provide obstetric assistance to sows or treat piglets. Prolonged inter-birth intervals can warrant obstetric assistance and consideration of pen features for optimum farrowing location (i.e., nest-site) that allows easy inspection and promotes good piglet survival is discussed in Section Quality of space—the importance of pen features and in depth elsewhere (4). Whilst it is important to factor into design the ability to help sows, it has been shown that FF sows may have less requirement for interventions; having reduced inter-birth intervals (3), showing lower incidence of pain-related behaviors during farrowing (46) and fewer post-partum health disorders (84). For treating piglets, mitigation measures include positioning of creep areas next to the passageway, with low pen divisions between passage and creep for easy piglet handling (85), automated opening of creep lids, or transparent lids, allowing quick inspection during checks without sudden opening causing piglets to startle and run out into the pen. Inspection windows in farrowing rooms could also reduce disturbance for more cursory checks. The ability to close the creep entrance prevents piglets escaping once gathered, thus reducing the need to enter the pen to collect piglets, often involving chasing to catch them (and promoting a negative HAR—Section Human-animal relationship). Closable creep fronts also aid with “creep training” [i.e., habituating piglets to using the creep (86)] and tasks like split suckling.

In German regulations it is a statutory requirement to ensure that the farrowing pen is designed “in such a way that no hazards can arise from the mother sow when catching or treating the piglets” (Table 1). As well as TC, other methods to help separate the stockpeople from the sow include lockable feeding stalls [e.g., (44)] and gates or walls between different pen areas [e.g., (40)]. People operating systems not allowing this physical separation emphasize the importance of developing positive HARs (Section Human-animal relationship) and gentling the sow prior to birth to allow safer practice post-partum (87, 88) when the sow's instincts are to be more maternally defensive (81).

#### Work conditions and room layout

The importance of ergonomics of fixtures and fittings are rarely investigated. The ease with which stockpeople can enter and exit the pen influences daily working routines, as well as safety. In their evaluation of five alternative systems, Gortz et al. (70) favored systems that had separate pen entry points rather than having to access through a feeder or across a creep. In a Swedish study of working routines in farrowing houses and their impact on musculo-skeletal problems (52), it was noted that functionality of certain features had significant impacts on work time and workload for different work elements. For example, the design of the gate locking system affected speed of gate opening and closing. From a workload perspective, it was considered disadvantageous to have to climb over a wall rather than walk through a gate during daily manure scraping. However, the gate locking mechanism and its ease of use influenced whether workers leant into pens to scrape muck or jumped over partitions.

For TC pens, the crate opening and closing equipment needs to be simple and not sensitive to the strength of the stockperson. Ideally it would only require use of one hand and the process of shutting the sow in the crate could be initiated from outside the pen. These ergonomic aspects are important not only to safeguard stockperson' health and welfare but also to help foster a less forceful HAR (89) (Section Human-animal relationship).

Having the ability to move sows in from multiple points can be useful but also adds complexity and therefore costs to any design. This is also the case for additional walkways between pens (i.e., to allow access from the back and the front). These allow full inspection of animals, as well as feeders/drinkers located away from a central passageway, avoiding entry into the pen but care should be taken to minimize disturbance to the sow or disruption of important stimuli to achieve good farrowing location (e.g., an enclosed nest-site—Section Quality of space—the importance of pen features). Despite the capital cost of discussed features, poor ergonomics impacts working routines, can impact stockperson health and productivity and thus have long-term cost implications (90).

The overall room layout and the optimum number of pens per room is influenced by working routines and batch size. When a Danish farmer was asked for recommendations regarding how many pens should be in one room, he replied no more than two rows of pens per room and no more than 24 pens in a room. Why? “*Sows are calm and quiet; staff can perform their routines efficiently and move on to the next batch… We can wean twice a week, start washing and cleaning a room without having to wait for more empty rooms. There is no disturbance, it is a comfortable place to work for staff and for pigs*” [Krogsgaard, (14)]. Whilst no scientific study has specifically investigated optimum room layout, this testimony about sow calmness is supported by scientific evidence relating to the “contagion effect” (Section Room design and pen number per room).

#### Multisystem comparison studies

Two major projects in the last 10 years have examined ease of management in alternative farrowing systems [Pro-SAU, Austria (64) and SEGES, Denmark (91)]. Heidinger et al. (64) examined duration of confinement period as well as manageability, and tested TC systems with different spatial footprints—three at 5.5 m^2^, one at 6.0 m^2^, and one at 7.4 m^2^. They found that confinement period had little effect on additional working time compared to a conventional farrowing crate (1.25–1.33 additional hours per sow per year from zero-confinement to confining 1 day before to 4 days after farrowing, respectively). However, pen-type was highly influential, ranging from 0.18 to 3.74 additional hours per sow per year, with the larger pen-type adding the most additional time.

Hansen (91) focused on various practical aspects of management (e.g., ease of handling, interventions, cleaning). All systems trialed apart from one were TC (with sows confined from ~3 days before until 5 days after farrowing). Five had partly solid floors and five had fully slatted floors, ranging in spatial footprint from 5.0 to 6.9 m^2^. Nest-building material (straw, jute sack) was supplied daily from transfer until farrowing. The study concluded that none of the brands were rated “good” or “very good” in all work parameters, but several of them achieved satisfactory results in several parameters. They commented that producers must decide on and prioritize their requirements for the pen since management routine and stockperson preferences vary from farm to farm. They also stressed the importance of pig producers visiting farms where the pens in question are used in large-scale production prior to making a choice.

### Human-animal relationship

As discussed in Section Room design and pen number per room layout can influence how well sows move in and out. Regardless of system, any impediment of movement, perceived or legitimate, will affect move-in times and could influence the relationship stockpeople have with the sow. If pigs are forcefully handled it creates a negative HAR and pigs quickly become fearful of humans (92), often avoiding or balking when being handled (93).

Developing a positive HAR is important for animal welfare and productivity. In the case of farrowing sows, fear of humans can indirectly influence crushing of piglets as fearful sows tend to change posture more frequently (94). There are links between negative HAR and a reduced number of piglets per sow per year and a positive HAR results in better growth and a lower level of stillbirths [reviewed in Coleman and Hemsworth (95)]. Sows that are less fearful of humans have better reproductive performance (i.e., total born and weaned piglets) (96, 97). Many scientific papers show that animals can develop neutral or positive perceptions of humans if proper actions are taken [reviewed by by Hemsworth (98)]. Repeated gentle contacts induce a decreased fear of humans and even an increased approach toward humans (92). Developing this good HAR prior to farrowing could help mitigate any potential safety issues post farrowing (99, 100) and careful/considered handling of piglets post-farrowing also reduces fearfulness (69, 101). Supporting the scientific evidence are reports by farmers about success factors for working with loose housed sows and the importance of stockperson' attitude toward the sows, their empathy toward them and the rewarding aspect of working with loose sows (18). In a case study in The Netherlands, the manager stated “*the majority see a better way of raising pigs as a very good improvement for their job… Our people are using more time to observe the animal behavior to prevent problems*.” (102). A small-scale study of stockpeople using TCs in Finland also stressed that “*free farrowing requires a better understanding of pig behavior and patience in work tasks from stockpeople*” (103).

### Stockperson training and attitude

There are a growing number of experienced stockpeople who work with non-confined sows during farrowing and lactation, including those who manage outdoor systems and those who have adopted indoor FF. Learning from those experienced in day-to-day management of these systems is seen as an important factor in encouraging successful adoption of alternatives (16). There is also evidence about what motivates farmers to make changes to enhance welfare (18). Researchers interviewed 12 pig farm owners and stockpeople from seven large commercial farms, showing that motivations were much higher if they were able to find others also changing aspects of their farm and to share experiences along the way. Networks and participatory experiences, either in the form of “stable schools” or their own established social networks, were highlighted as positively supporting change and generating innovation. These authors also cite that the feeling of control and being consulted on projects were important to stockpeople, which influenced their connection and engagement with the project. Stockpeople liked the idea of being early adopters, involved in innovation and having an influence on decisions (18). In Finland, stockpeople urged those making decisions to consider their work as a whole, because working with FF sows takes more time and any changes in system/practice affects the stockperson's ability to do their job well and safely. They noted that safe and smooth work increases their work motivation and job satisfaction (103). Thus, consulting stockpeople in aspects of pen design that affect their day-to-day working conditions makes sense.

The importance of having “*strict procedures in place from the simplest of tasks to the more complex*” when working with loose sows was something emphasized in case studies (102). This ensures there is minimal disturbance, particularly around new mothers and litters, encouraging good maternal behavior, reducing requirements for intervention. It is also important because not all farms will have a highly skilled and engaged work force. Stockpeople could be transient and require detailed instructions. A more predictable system of management, consisting of a combination of specific routines, is important around the time of farrowing (88).

The evidence cited thus indicates that approaching the transition to FF and/or TC with an open mind and understanding that there may need to be adaptability in established routines for stockpeople and animals, are likely to be important success factors.

### Considerations for the farmer/owner

Whilst farmers may also be stockpeople, they have a more specific role in strategic decision making and management of the overall business. The factors which need to be taken into consideration in this context are the effects of key farrowing system decisions on economics and productivity outcomes.

#### Economics

Investing in new farrowing accommodation will require budgeting for various costs. Costs will depend on which system is chosen; outdoor and certain group housing systems are generally regarded as low-cost alternatives, whereas individual pens with sophisticated pen features are more expensive. The main costs relate to increased space requirements for most alternatives. However, costs of any farrowing system are not only dependent on capital investment but also on running costs, performance and efficiency (104).

The major areas in which costs of alternative systems may differ from farrowing crates are capital costs of pen structure, building space allowance and the associated costs of heating/cooling increased airspace (depending on room area), substrate provision, potential for piglet mortality differences and labor requirements (104). In addition, increasing pen size to meet animals' behavioral needs is also likely to increase emissions (Section Environmental impacts), something under regulatory control. Therefore, farmers may not be able to expand their pig buildings without further investment in technology to reduce environmental impact or a reduction in herd size. The first option leads to increasing cost and the second option leads to decreasing income. Consequently, both solutions are unfavorable for farm economy.

AHDB Pork (105) completed an economic evaluation of alternative systems for the UK industry using established costings models developed by InterPig. They concluded that “*Based on the evidence currently available, when taking account of likely changes to physical performance and costings, we expect the cost of production for GB indoor herds installing alternative farrowing systems to increase by 3–8 p/kg deadweight depending on the chosen pen design's footprint and the mortality achieved. Even for those producers who might achieve comparable pre-weaning mortality levels, costs are likely to rise by 3–5 p/kg*.” Despite this conclusion the UK government has signaled their intention to reduce confinement for farrowing sows as expressed in their Pig Welfare Codes (106). The Danish government also signaled its intent to move toward reduced confinement with the introduction of the “Better Welfare” label, a joint government and industry initiative (107).

Previous modeling exercises by Guy et al. (104) compared one TC system occupying the same spatial footprint as a conventional crate (4.3 m^2^) and two FF systems of 6.0 and 8.9 m^2^. They concluded that (assuming equitable pig performance across modeled systems) there would be a higher production cost for non-crate systems by 1.6, 1.7 and 3.5%, respectively. They also examined cost-neutral or profit-making scenarios, including modeling improvements in weaning weight, which has been demonstrated by a number of studies (9, 45–47). The ProSAU project (64) calculated higher capital investment costs with TC pens (assuming equitable pig performance across all pen types studied) of +28.3% for a TC of 5.5 m^2^ compared to the farrowing crate of 4.0 m^2^. They also calculated additional labor costs of ~€10 per sow per year for the TC. Few studies have followed piglets through to finishing to determine if there are advantages in days to slaughter and lifetime daily gain. To the best of our knowledge only one study has reported significant long-term benefits in litters from loose-housed sows (11). Some studies have followed piglets immediately post-weaning, highlighting lower salivary stress biomarkers for piglets reared in some alternatives but not all [e.g., (108) compared two TCs against permanent crating]. Being better adapted to weaning is one advantage highlighted for GH systems [for review see (22)] and quantifying the possible longer term economic advantage of this would be a useful exercise.

Other benefits that could offset the costs, but are not widely evaluated, include rebreeding efficiency, with one study showing an increase in subsequent litter size after initially farrowing in pens where the sow was loose (109). There are emerging studies on how systems that allow both the sow and piglets greater freedom of movement and behavioral expression can improve the resilience of piglets in the longer-term [e.g., by changing immune competence, accelerating the maturation of gut microbiota—for review see (110)] which might impact health and welfare, and thus economic performance. However, evidence on the lifetime consequences of alternative farrowing systems remains sparse. Any performance advantages will help offset the capital costs, reducing cost of production, especially if a price premium cannot be achieved. Some countries offer subsidies for such initiatives. Sweden's “suggpengar” subsidizes running costs (111). Denmark's “Farestalde” subsidizes capital investment (112). Various assurance schemes pay premiums for welfare improvements, with some including changes to farrowing accommodation in their standards (14, 113, 114).

#### Performance

A well-designed and managed system without permanent crating can achieve the same performance as conventional farrowing crates. This has been demonstrated in research [e.g., (8) for review] and, perhaps more importantly, under commercial conditions [e.g., (14, 38, 40, 115)]. Review articles attempting to summarize performance information of alternative farrowing systems have cited a number of important caveats (3, 5–8); specifically that summarizing can result in loss of details of particular studies that might contribute to explaining performance outcomes. For example, breed differences, sow parity and previous experiences, the similarity between dry sow accommodation and farrowing accommodation have all been shown to influence piglet survival. In addition, when comparing housing systems, it should be the performance of the system that is compared, so including all piglets born in the system with analysis at the batch-level to take into account piglet movements due to cross-fostering and use of nurse sows. This is particularly important when considering systems with hyperprolific sows. However, when analyzing information at batch-level, information about the individual litter is lost and supplementary analysis of piglet mortality at sow level can be helpful to learn about effects of things like litter size, parity, age at death, etc. (71).

#### The learning curve associated with a new system

When changing systems, stockperson and sow experience can impact performance outcomes during a transition period. There is evidence of a learning curve when new systems are installed on farms. Stockperson' experience is a factor. In a study where two farms ran the PigSAFE FF system and farrowing crates on their respective sites, there were site differences in how well systems performed (47). Site 1 returned live-born mortality <9% for both crates and PigSAFE, whilst on Site 2 mortality in PigSAFE was numerically higher than crates. The Site 1 farmer had previously managed outdoor pigs and was experienced with loose sows, whereas the Site 2 farmer had only ever worked with sows in farrowing crates. Inspection of the data showed batch effects with piglet survival improving as the Site 2 farmer learned how to manage the system. Similar results were shown by farmers in a Norwegian commercial uptake trial of the SowComfort pen (40). Live-born mortality decreased by 3%-points over five batches and remained consistent once routines had been established and farmers had learnt the system. In a survey of Danish farmers (18) a number of stockpeople mentioned how the 1st year was chaotic, something they return to (during interviews): “*A year ago, everyone was new, the previous employees were no longer here, only one had experience—but not about loose sows in the farrowing unit. The owner's word for it* ‘*We had no history*.”'

Sows also have to get used to a new system. Studies show that sows that farrow in the same farrowing environment from 1st to 2nd parity perform better (lower crushing mortality) than those moving between systems (79, 109). This is particularly important as farmers think about transitioning to new systems. They may want to initially try a few pens, meaning that pigs and stockpeople will be swapping between crates and alternatives. The results from King et al. (79, 109) provide the first scientific evidence of something that farmers suspected was influencing performance. On a Dutch farm the manager noted problems with sows swapping between crates and an alternative (TC) system, commenting that sometimes, when gilts experienced TC for the first litter, they refused to enter a crate for their next. Consequently, they had to be moved or they stopped eating. Although these farmers thought swapping between systems likely affected performance they did not formally study it (102).

## Tier 3 stakeholder needs and considerations

The uptake of new farming systems is not only influenced by the preferences of those who directly work with them, but also by the wider legislative and market environment in which they must exist. For this reason, the views of stakeholders representing the wishes of wider civil society, as commonly translated through politicians and retailers, are important to take into account. Whilst it is the case that the cost and quality of the product still exert an over-riding influence on purchasing decisions (116) increasing concerns for sustainable production are apparent (117) as evidenced by the growth in both public and private labeling schemes based on method of production criteria. Sustainable production can encompass many considerations (118), but in the context of farrowing systems the primary focus is on respect for animals and their welfare, and on protection of the environment through reducing carbon footprint and polluting emissions.

### The market for meat

One way in which consumers can make their preferences regarding production systems known, and consequently influence farming practice, is through their purchasing decisions. This was clearly shown in the case of table eggs, where the introduction of a mandatory EU labeling scheme identifying whether or not eggs were produced in a cage system has been accompanied by an increase in the use of alternative systems (119). There is currently no comparable “single issue” labeling for pig-meat which focusses on the absence of a “cage” in production. Consumers must therefore identify through their own efforts which of the many available voluntary labeling schemes include this criterion amongst many other standards relating to animal welfare, and often also food safety and environmental issues (113, 114). The use of such complex schemes has two important consequences—one is that it confuses consumers and dilutes the impact of the “cage-free” message, and the other is to increase the product price to cover all of the diverse production requirements involved. As discussed in Section Economics, a simple ban on farrowing crates has been estimated to increase production cost by 3–8 p/kg deadweight [~2–5% at the time of the UK-based study—(105)]. However, it has been estimated that the increase in production cost to meet all requirements of a Danish voluntary labeling scheme for pigs is ~26% (120). The effect on purchase price may be significantly greater since not all of the carcass weight translates into premium-priced products; it is known that only a percentage of the carcass (the more desirable cuts) can be sold at a premium under labeling schemes. When sold as a “high welfare” package under current EU voluntary labeling schemes, price premia in the shops as high as 42–55% have been reported for pig-meat products (121). This obviously impacts on the likely volume of sales, and hence the market impact on production practice. An EU-wide survey on animal welfare issues in 2016 (122) reported that while 59% of citizens said that they were willing to pay more for animal-welfare friendly product, only 14% indicated that they would pay a premium of 11% or more. Furthermore, it is well-demonstrated that such intention statements are often not translated into purchasing action (121). These considerations suggest that, under current market conditions, the probability that consumers will influence a transition away from farrowing crates is limited.

### Environmental impacts

Other factors are increasingly part of the political decision-making process—namely the influences of production choices on the environment (123). However, there is a lack of research regarding the environmental impacts of alternative farrowing systems and often modeling predictions are used to discuss impacts.

Alternative systems require extra space, but additional building materials per sow place used for construction of new systems will have little impact. Life Cycle Analyses of pig-meat production indicate that for all major impact categories (e.g., Global Warming Potential, Acidification Potential, Eutrophication Potential), the contribution of initial building costs is greatly outweighed by the ongoing consequences of feed use and manure management (124). Thus, if a change in farrowing system has an ongoing negative effect on the level of production (sow feed use per piglet sold), this will increase the environmental footprint of the product produced. However, whilst there are concerns regarding increased piglet mortality, this need not be the case for alternative farrowing systems which are well-designed and operated (Section Performance).

Overall building design, as well as specific pen features and design choices like flooring and slurry system all impact emissions. The increase in space per pen will result in increased ammonia emissions if the slurry surface in the pen also increases (125). If the slurry surface stays the same but the solid floor increases and it remains unfouled, ammonia emissions could stay the same. However, in TC-pens sows dung and urinate in one position when confined but often choose to dung and urinate elsewhere in the pen when loose (51, 126). To maintain hygiene stock workers would have to clean out more frequently or pens need a larger area of slatted floor (126) which increases ammonia emissions (125, 127). Emissions of methane are less related to the emission surface and more to the volume of bulk liquid, the storage temperature of slurry and the frequency of its removal (128). However, use of substantial amounts of straw increases the dry matter content of slurry which increases the formation and production of methane. Slurry systems that enable frequent removal of slurry (e.g., scraper and back flushing systems) and a very low amount of bulk liquid have been shown to lower the odor (129) and methane emissions (130).

### Societal acceptability

Citizens' concerns about restrictive housing conditions of farm animals have increased in the last years, as shown by the recent success of the ECI “End the Cage Age” aiming at prohibiting all cage systems, which collected over a million signatures across 18 EU member states. Most studies show that the use of farrowing crates does not have societal support (131, 132). Boogaard et al. (131) conducted visits of conventional and organic pig farms in the Netherlands and Denmark with citizen panels. Regarding farrowing systems, respondents valued freedom of movement, wanting to see “*unfixed sows in spacious farrowing pens*”. In response to such concerns, there have been voluntary industry initiatives for change. For example, in Denmark in 2011 the industry set targets for 10% of the breeding herd to be loose lactating by 2021 (83) and the “Better Welfare” label is a joint government/industry initiative and includes partners from the food chain [English—Bedre Dyrevelfærd (bedre-dyrevelfaerd.dk)]. The goal of 10% of lactating sows to be loose by 2021 has not been reached. However, the initiative has recently been followed up by another industry initiative announcing that from 2023 all sows in new-build facilities will be loose-housed (133). Outside of the EU, Vandresen and Hotzel (132) investigated Brazilian citizens' attitudes toward farrowing crates, loose pens, and outdoors. They reported a low support for farrowing crates based on concerns over sows' freedom of movement, behavioral freedom and naturalness, even with the associated risk of piglet mortality. These authors have also suggested that social acceptability of loose housing may be undermined by use of TC and the possibility of restraint. Furthermore, as with the partial gestation stall ban, there are concerns about how to audit the confinement period to ensure maximum permitted restraint periods are adhered to. Hence, there may be future challenges for early adopters if they install transition systems that consumers might find unacceptable or are unlikely to be compliant with policy changes. Similar challenges have been evident in the egg-industry as battery cages were prohibited in Europe in 2012 but furnished, enriched or colony cages were permitted (European Union Council Directive 1999/74/EC). Egg producers believed and invested in systems they expected to be future-proof, however after changing from battery cages to enriched cages it turned out not to be the case. “A cage is still a cage” was the reaction of many NGOs and consumers when investment was made in enriched cages (19), which are now precluded from many markets and under threat with the ECI. This mindset may slow down or prevent possible commercial uptake of TC systems.

## Conclusion and knowledge gaps

This review has discussed the importance of different aspects when transitioning from farrowing crates to alternative systems and how the decisions made impact on multiple stakeholders. It is recognized that the pivotal starting point for those investing in new systems is the spatial footprint per sow place. Specified space criteria are also favored by legislators, as they offer simple compliance audits. However, generalizing on space is problematic because success of any system depends more on the quality of space, including spatial dimensions, pen features, overall room layout, as well as the ability of animals to use available space. How space impacts their welfare is determined by their ability to use the space to perform fundamental behaviors including: turning around, fully expressing inherently motivated behaviors such as nest building, engaging in successful mother-offspring interactions and differentiating functional areas. There is also the over-riding effect of stockperson' “quality.” What is evident, however, is that certain spatial footprints (≤5.5 m^2^) can be considered too small to operate fully FF. Consequently, such footprints require use of TC and most likely will require a fully slatted floor, which has implications for both societal acceptability and environmental regulations. More generous footprints (e.g., ≥6.5 m^2^) have greater potential to be operated as true FF but are heavily dependent on specific pen design and confidence of stockpeople to operate without the safety net of TC. As such, EFSA's Opinion (3) regarding TCs is that they cannot be advised “as a step in a farm's transition from using farrowing crates to farrowing pens, unless the size of the temporary farrowing crate system is the same as that of the future free farrowing pen.”

Given the over-riding influence of the human-animal relationship in the success of alternative systems, the needs and training of stockpeople must be fully taken into account. There must be an understanding that the variety of alternative systems available may mean different protocols are required for different systems and developing routines and stable performance will involve a transition period. Consistency over this period is also important for the animals.

Whilst there has been research into alternative farrowing systems for over 40 years, there remain important gaps in knowledge that should be considered to help inform the transition from crates to free farrowing:

- There is a lack of information on pen details described in publications, making it difficult to compare designs and to provide specific recommendations.
- Despite much work on animal spatial requirements to perform functional behaviors, empirical evidence is lacking on space required for sow turning and grouping/clustering of piglets, to minimize crushing, and the interaction with other nest features.
- Little attention has been given to overall room design, layout and number of pens per room and its impact on welfare, animal behavior, human behavior and their relationship.
- Although critical for a successful transition to loose housing, peer review evidence of the farmers' point of view on the different aspects of sow housing conditions (pen and room features) as well as working with loose sows is mostly missing.
- Changing to loose housing needs to be associated with economically viable pig-meat production. Whilst data on piglet mortality and growth rate of piglets during lactation in loose housing are reasonably abundant, data on long-term performance (and other outcomes) to finishing, as well as the potential impacts on sow longevity, remain sparse.
- There is a lack of empirical data on the environmental impacts of alternative systems which is crucial in order to make more informed decisions about the likely trade-offs between welfare, costs and environment to achieve sustainable pig production.

## Author contributions

The conceptual work, review, and manuscript drafting was led by EB with support from SE, SG, GI, and VM. All authors contributed to the article and approved the submitted version.

## Funding

EB was supported by the Scottish Government's Rural and Environment Science and Analytical Services Division (RESAS SRUC-A3-5). VM was partly funded by the Danish Pig Levy Foundation. GI was supported by grant MZE-RO0718 from the Ministry of Agriculture of the Czech Republic.

## Conflict of interest

Author VM worked for SEGES Innovation. The aim of SEGES Innovation is to safeguard the interests of the Danish farmers. The remaining authors declare that the research was conducted in the absence of any commercial or financial relationships that could be construed as a potential conflict of interest.

## Publisher's note

All claims expressed in this article are solely those of the authors and do not necessarily represent those of their affiliated organizations, or those of the publisher, the editors and the reviewers. Any product that may be evaluated in this article, or claim that may be made by its manufacturer, is not guaranteed or endorsed by the publisher.
